# Ethyl 4-[({[(6-chloro­pyridin-3-yl)meth­yl](meth­yl)amino}(4-fluoro­anilino)­methyl­idene)amino]-3-phenyl-2-sulfanyl­idene-2,3-dihydro-1,3-thia­zole-5-carboxyl­ate

**DOI:** 10.1107/S1600536812026311

**Published:** 2012-06-30

**Authors:** Hai-Feng He, Hong-Wu He, Ying Liang, Zi-Wen Yang

**Affiliations:** a Hubei Biopesticide Engineering Research Center, Hubei Academy of Agricultural Science, Wuhan 430064, People’s Republic of China; b Key Laboratory of Pesticide & Chemical Biology of the Ministry of Education, Central China Normal University, Wuhan 430079, People’s Republic of China.

## Abstract

In the title compound, C_26_H_23_ClFN_5_O_2_S_2_, the mean plane of the guanidine fragment makes dihedral angles of 58.94 (13), 78.37 (17) and 50.76 (15)°, respectively, with the attached thia­zole, pyridine and phenyl rings. The crystal structure features N—H⋯S and C—H⋯O hydrogen bonds and weak π–π stacking inter­actions [centroid–centroid separation = 3.7702 (17) Å]. The terminal methyl group of the eth­oxy­carbonyl group is disordered over two orientations in a 0.836 (10):0.164 (10) ratio.

## Related literature

For further synthetic details and background to thia­zolo­pyrimidines, see: Liang *et al.* (2007 ▶).
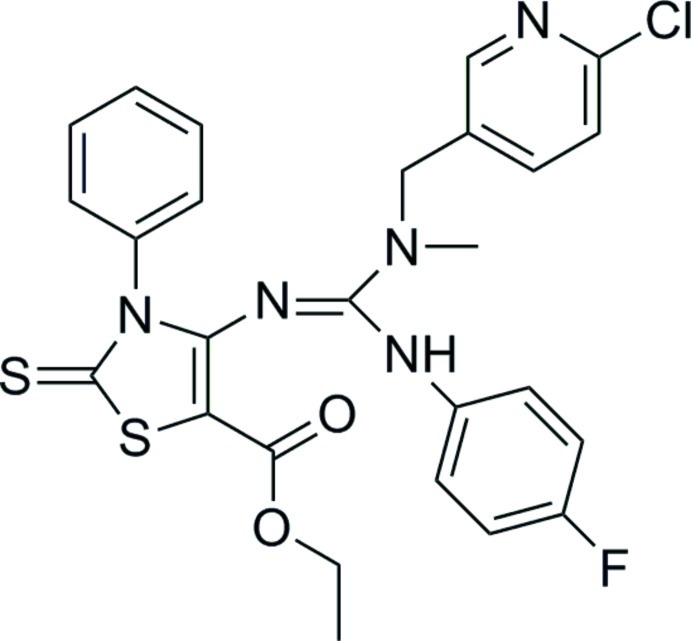


## Experimental

### 

#### Crystal data

C_26_H_23_ClFN_5_O_2_S_2_
*M* *_r_* = 556.06
Monoclinic, 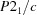

*a* = 9.6931 (5) Å
*b* = 24.5636 (12) Å
*c* = 11.7095 (6) Å
β = 103.745 (1)°
*V* = 2708.2 (2) Å^3^
*Z* = 4
Mo *K*α radiation
μ = 0.34 mm^−1^
*T* = 298 K
0.20 × 0.10 × 0.06 mm

#### Data collection

Bruker SMART APEX CCD diffractometer
Absorption correction: multi-scan (*SADABS*; Sheldrick, 2001 ▶) *T*_min_ = 0.936, *T*_max_ = 0.980
23442 measured reflections
5326 independent reflections
3569 reflections with *I* > 2σ(*I*)
*R*_int_ = 0.097

#### Refinement

*R*[*F* ^2^ > 2σ(*F* ^2^)] = 0.061
*wR*(*F* ^2^) = 0.172
*S* = 1.00
5326 reflections
350 parameters
23 restraints
H atoms treated by a mixture of independent and constrained refinement
Δρ_max_ = 0.50 e Å^−3^
Δρ_min_ = −0.40 e Å^−3^

### 

Data collection: *SMART* (Bruker, 2000 ▶); cell refinement: *SAINT* (Bruker, 2000 ▶); data reduction: *SAINT*; program(s) used to solve structure: *SHELXS97* (Sheldrick, 2008 ▶); program(s) used to refine structure: *SHELXL97* (Sheldrick, 2008 ▶); molecular graphics: *SHELXTL* (Sheldrick, 2008 ▶); software used to prepare material for publication: *SHELXTL*.

## Supplementary Material

Crystal structure: contains datablock(s) global, I. DOI: 10.1107/S1600536812026311/hb6808sup1.cif

Structure factors: contains datablock(s) I. DOI: 10.1107/S1600536812026311/hb6808Isup2.hkl

Additional supplementary materials:  crystallographic information; 3D view; checkCIF report

## Figures and Tables

**Table 1:** Hydrogen-bond geometry (Å, °)

*D*—H⋯*A*	*D*—H	H⋯*A*	*D*⋯*A*	*D*—H⋯*A*
N3—H3*A*⋯S2^i^	0.86 (3)	2.65 (3)	3.443 (3)	153 (3)
C7—H7*B*⋯O1^ii^	0.96	2.51	3.282 (4)	138

Symmetry codes: (i) 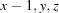; (ii) 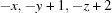.

**Fig. 1.**
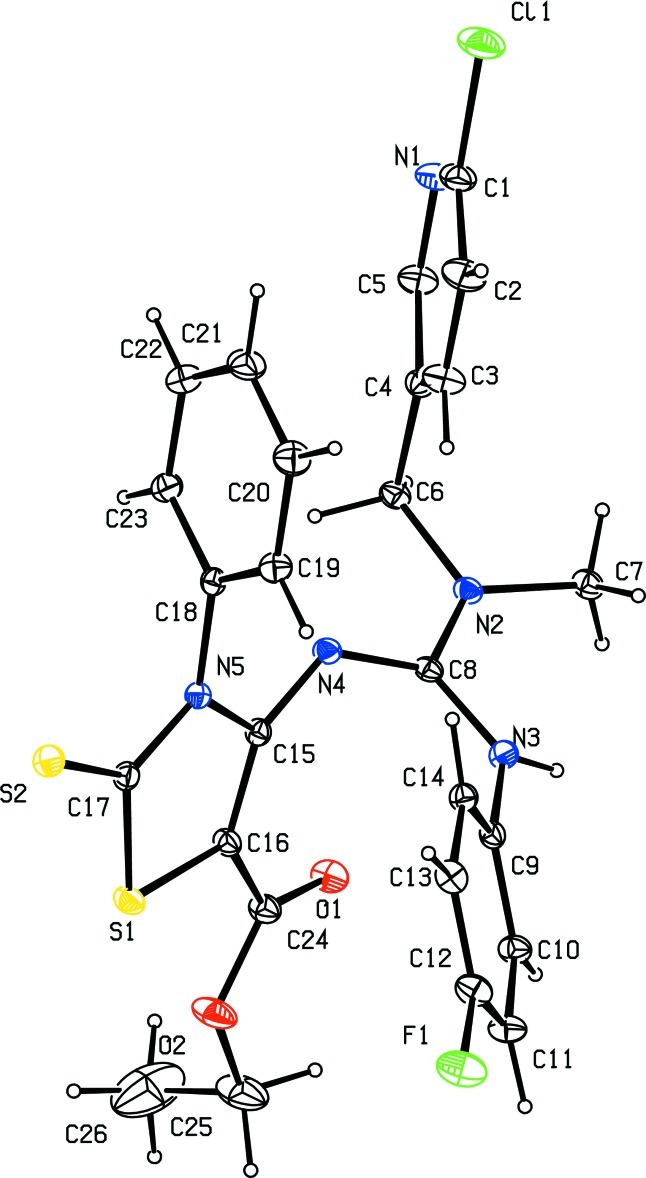
The structure of (I), showing 50% probability displacement ellipsoids.

**Fig. 2.**
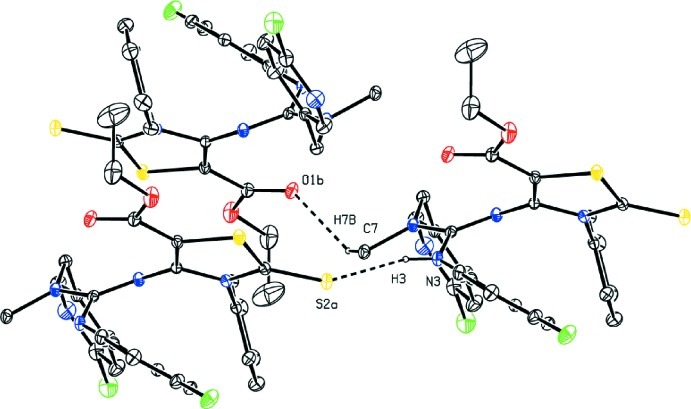
Crystal Packing diagram of (I). Hydrogen bonds are shown as dashed lines.

## supplementary crystallographic information

### Comment

Recently, we have developed a new and versatile annulation process, which proceeded smoothly under mild conditions *via* a tandem aza-wittig and cyclization reaction (Liang *et al.*, 2007), to synthesize new thiazolo[4,5-*d*]pyrimidine derivatives with possible herbicidal activities. In this paper, we report the structure of the intermediate guanidine derivative, (I) (Fig. 1).

In the molecule of the title compound, (I), the mean plane of the guanidine system is nearly coplannar. The three aryl groups are roughly twisted from the central guanidine system, the dihedral being 58.94 (13)°(thiazole), 78.37 (17)° (pyridine) and 50.76 (15)° (*p*-fluorophenyl) respectively, and the thiazole ring is nearly planar with the ethyloxyacyl group. The crystal packing is stabilized by C—H···O and C—H···S hydrogen bonds (Table 1) and weak π-π stacking interactions [*Cg*1···*Cg*3^i^ = 3.7702 (17) Å; symmetry code: (i) *x*, *y*, *z*]. The atom C26 was disorder.

### Experimental

To a solution of the iminophosphorane (1 mmol) in dry CH_2_Cl_2_(15 ml) was added 4-fluorophenyl isocyanate (1.1 mmol) under an N_2_ atmosphere at room tempreture. After the reaction mixture was allowed to stand for 5–12 h, the solvent was removed under reduced pressure, then Et_2_O and petroleum ether were added to precipitate the side product triphenylphosphine oxide which was then removed by filtration. Subsequent removal of the solvent gave the corresponding carbodiimide, which was used directly without further purification. To a solution of the carbodiimide in ethanol(15 ml) was added 1-(6-chloropyridin-3-yl)-*N*-methyl methanamine (1.1 mmol) and a catalytic amount of sodium ethoxide in ethanol. After the mixture had been stirred for 4 h at 303 K, the solution was concentrated and the residue was recrystallized from CH_3_CN solution to give colorless blocks of the title compound, (I), after one week.

### Refinement

All H-atoms bound to carbon were refined using a riding model with d(C—H) = 0.93 Å, *U*_iso_=1.2*U*_eq_ (C) for aromatic 0.98 Å, *U*_iso_ = 1.2*U*_eq_ (C) for CH 0.96 Å, *U*_iso_ = 1.5*U*_eq_ (C) for CH3 H atoms.

### Crystal data

**Table:**

C_26_H_23_ClFN_5_O_2_S_2_	*F*(000) = 1152
*M**_r_* = 556.06	*D*_x_ = 1.364 Mg m^−^^3^
Monoclinic, *P*2_1_/*c*	Mo *K*α radiation, λ = 0.71073 Å
*a* = 9.6931 (5) Å	Cell parameters from 4840 reflections
*b* = 24.5636 (12) Å	θ = 2.3–25.7°
*c* = 11.7095 (6) Å	µ = 0.34 mm^−^^1^
β = 103.745 (1)°	*T* = 298 K
*V* = 2708.2 (2) Å^3^	Needle, colourless
*Z* = 4	0.20 × 0.10 × 0.06 mm

### Data collection

**Table:**

Bruker SMART APEX CCD diffractometer	5326 independent reflections
Radiation source: fine-focus sealed tube	3569 reflections with *I* > 2σ(*I*)
Graphite monochromator	*R*_int_ = 0.097
φ and ω scans	θ_max_ = 26.0°, θ_min_ = 1.7°
Absorption correction: multi-scan (*SADABS*; Sheldrick, 2001)	*h* = −11→11
*T*_min_ = 0.936, *T*_max_ = 0.980	*k* = −30→27
23442 measured reflections	*l* = −14→14

### Refinement

**Table:**

Refinement on *F*^2^	Primary atom site location: structure-invariant direct methods
Least-squares matrix: full	Secondary atom site location: difference Fourier map
*R*[*F*^2^ > 2σ(*F*^2^)] = 0.061	Hydrogen site location: inferred from neighbouring sites
*wR*(*F*^2^) = 0.172	H atoms treated by a mixture of independent and constrained refinement
*S* = 1.00	*w* = 1/[σ^2^(*F*_o_^2^) + (0.0958*P*)^2^] where *P* = (*F*_o_^2^ + 2*F*_c_^2^)/3
5326 reflections	(Δ/σ)_max_ < 0.001
350 parameters	Δρ_max_ = 0.50 e Å^−^^3^
23 restraints	Δρ_min_ = −0.40 e Å^−^^3^

### Special details

**Table:**

Geometry. All e.s.d.'s (except the e.s.d. in the dihedral angle between two l.s. planes) are estimated using the full covariance matrix. The cell e.s.d.'s are taken into account individually in the estimation of e.s.d.'s in distances, angles and torsion angles; correlations between e.s.d.'s in cell parameters are only used when they are defined by crystal symmetry. An approximate (isotropic) treatment of cell e.s.d.'s is used for estimating e.s.d.'s involving l.s. planes.
Refinement. Refinement of *F*^2^ against ALL reflections. The weighted *R*-factor *wR* and goodness of fit *S* are based on *F*^2^, conventional *R*-factors *R* are based on *F*, with *F* set to zero for negative *F*^2^. The threshold expression of *F*^2^ > σ(*F*^2^) is used only for calculating *R*-factors(gt) *etc*. and is not relevant to the choice of reflections for refinement. *R*-factors based on *F*^2^ are statistically about twice as large as those based on *F*, and *R*- factors based on ALL data will be even larger.

### Fractional atomic coordinates and isotropic or equivalent isotropic displacement parameters (Å^2^)

**Table:**

	*x*	*y*	*z*	*U*_iso_*/*U*_eq_	Occ. (<1)
C1	0.2452 (4)	0.72915 (14)	0.9071 (4)	0.0648 (10)	
C2	0.2408 (5)	0.69171 (16)	0.8193 (3)	0.0750 (11)	
H2	0.2568	0.7017	0.7470	0.090*	
C3	0.2115 (5)	0.63856 (14)	0.8432 (3)	0.0682 (10)	
H3	0.2079	0.6119	0.7862	0.082*	
C4	0.1875 (3)	0.62471 (12)	0.9506 (2)	0.0441 (7)	
C5	0.1929 (4)	0.66619 (14)	1.0298 (3)	0.0652 (10)	
H5	0.1753	0.6574	1.1023	0.078*	
C6	0.1574 (3)	0.56709 (12)	0.9822 (3)	0.0470 (8)	
H6A	0.2467	0.5477	1.0065	0.056*	
H6B	0.1137	0.5679	1.0486	0.056*	
C7	−0.0833 (3)	0.55378 (12)	0.8587 (3)	0.0499 (8)	
H7A	−0.1147	0.5606	0.7759	0.075*	
H7B	−0.0936	0.5863	0.9014	0.075*	
H7C	−0.1396	0.5252	0.8806	0.075*	
C8	0.1212 (3)	0.50286 (11)	0.8189 (2)	0.0386 (7)	
C9	0.0566 (3)	0.44719 (12)	0.6372 (2)	0.0411 (7)	
C10	−0.0089 (3)	0.39778 (13)	0.6085 (3)	0.0515 (8)	
H10	−0.0700	0.3841	0.6519	0.062*	
C11	0.0165 (4)	0.36822 (14)	0.5142 (3)	0.0620 (9)	
H11	−0.0294	0.3353	0.4921	0.074*	
C12	0.1103 (4)	0.38872 (15)	0.4550 (3)	0.0602 (9)	
C13	0.1761 (4)	0.43763 (14)	0.4815 (3)	0.0541 (8)	
H13	0.2397	0.4504	0.4395	0.065*	
C14	0.1462 (3)	0.46790 (13)	0.5720 (2)	0.0452 (7)	
H14	0.1864	0.5022	0.5891	0.054*	
C15	0.3352 (3)	0.45864 (12)	0.8084 (2)	0.0383 (7)	
C16	0.3268 (3)	0.40313 (12)	0.8165 (2)	0.0436 (7)	
C17	0.5342 (3)	0.43200 (13)	0.7403 (2)	0.0434 (7)	
C18	0.4900 (3)	0.53030 (12)	0.7543 (2)	0.0408 (7)	
C19	0.4673 (4)	0.55250 (14)	0.6438 (3)	0.0537 (8)	
H19	0.4318	0.5311	0.5778	0.064*	
C20	0.4975 (4)	0.60657 (15)	0.6316 (4)	0.0699 (10)	
H20	0.4817	0.6219	0.5570	0.084*	
C21	0.5505 (4)	0.63778 (15)	0.7284 (4)	0.0742 (11)	
H21	0.5715	0.6743	0.7198	0.089*	
C22	0.5730 (4)	0.61527 (15)	0.8390 (4)	0.0724 (11)	
H22	0.6086	0.6368	0.9048	0.087*	
C23	0.5434 (4)	0.56148 (13)	0.8531 (3)	0.0552 (8)	
H23	0.5591	0.5462	0.9278	0.066*	
C24	0.2234 (4)	0.37141 (13)	0.8606 (3)	0.0521 (8)	
O2	0.2516 (3)	0.31823 (10)	0.8546 (3)	0.0856 (9)	
C25	0.1581 (6)	0.27977 (19)	0.8952 (5)	0.1149 (18)	
H25A	0.1402	0.2486	0.8429	0.138*	0.836 (10)
H25B	0.0679	0.2971	0.8938	0.138*	0.836 (10)
H25C	0.1139	0.2972	0.9517	0.138*	0.164 (10)
H25D	0.2123	0.2488	0.9332	0.138*	0.164 (10)
C26	0.2260 (11)	0.2612 (4)	1.0182 (5)	0.212 (5)	0.836 (10)
H26A	0.3246	0.2534	1.0244	0.317*	0.836 (10)
H26B	0.1791	0.2289	1.0357	0.317*	0.836 (10)
H26C	0.2175	0.2893	1.0730	0.317*	0.836 (10)
C26'	0.035 (2)	0.2639 (11)	0.7926 (17)	0.117 (16)	0.164 (10)
H26D	−0.0306	0.2937	0.7743	0.175*	0.164 (10)
H26E	−0.0127	0.2326	0.8140	0.175*	0.164 (10)
H26F	0.0711	0.2554	0.7251	0.175*	0.164 (10)
Cl1	0.28382 (14)	0.79686 (4)	0.88166 (13)	0.0998 (4)	
F1	0.1395 (3)	0.35853 (9)	0.36600 (19)	0.0894 (8)	
N1	0.2212 (4)	0.71815 (12)	1.0114 (3)	0.0754 (9)	
N2	0.0652 (3)	0.53743 (10)	0.8866 (2)	0.0424 (6)	
N3	0.0259 (3)	0.47731 (11)	0.7322 (2)	0.0455 (6)	
N4	0.2593 (2)	0.50043 (10)	0.8382 (2)	0.0430 (6)	
N5	0.4550 (2)	0.47396 (10)	0.76883 (18)	0.0393 (6)	
O1	0.1278 (3)	0.38916 (9)	0.8991 (2)	0.0607 (6)	
S1	0.46244 (9)	0.37121 (3)	0.76844 (7)	0.0518 (3)	
S2	0.67662 (9)	0.43702 (4)	0.68492 (7)	0.0546 (3)	
H3A	−0.059 (4)	0.4739 (14)	0.742 (3)	0.066*	

### Atomic displacement parameters (Å^2^)

**Table:**

	*U*^11^	*U*^22^	*U*^33^	*U*^12^	*U*^13^	*U*^23^
C1	0.063 (2)	0.0369 (19)	0.099 (3)	−0.0057 (16)	0.029 (2)	−0.0006 (19)
C2	0.104 (3)	0.050 (2)	0.083 (3)	−0.002 (2)	0.048 (2)	0.0081 (19)
C3	0.101 (3)	0.045 (2)	0.069 (2)	−0.0017 (19)	0.039 (2)	−0.0037 (17)
C4	0.0437 (18)	0.0404 (17)	0.0499 (16)	0.0029 (14)	0.0146 (13)	−0.0015 (14)
C5	0.088 (3)	0.049 (2)	0.064 (2)	−0.0016 (19)	0.029 (2)	−0.0085 (17)
C6	0.056 (2)	0.0412 (18)	0.0461 (16)	0.0055 (14)	0.0157 (14)	−0.0039 (13)
C7	0.051 (2)	0.0468 (19)	0.0588 (18)	0.0063 (15)	0.0255 (15)	−0.0028 (15)
C8	0.0443 (18)	0.0302 (15)	0.0449 (15)	−0.0002 (13)	0.0180 (13)	0.0039 (12)
C9	0.0364 (16)	0.0434 (18)	0.0443 (15)	0.0054 (13)	0.0112 (13)	−0.0021 (13)
C10	0.054 (2)	0.047 (2)	0.0597 (19)	−0.0017 (15)	0.0237 (16)	−0.0079 (15)
C11	0.078 (3)	0.046 (2)	0.066 (2)	−0.0057 (18)	0.0252 (19)	−0.0135 (17)
C12	0.079 (3)	0.056 (2)	0.0527 (18)	0.0077 (19)	0.0290 (18)	−0.0054 (16)
C13	0.060 (2)	0.058 (2)	0.0489 (18)	0.0070 (17)	0.0218 (15)	0.0046 (16)
C14	0.0454 (18)	0.0449 (18)	0.0448 (16)	−0.0003 (14)	0.0097 (13)	0.0005 (13)
C15	0.0347 (16)	0.0417 (17)	0.0397 (14)	0.0008 (13)	0.0111 (12)	−0.0006 (12)
C16	0.0415 (17)	0.0391 (18)	0.0531 (17)	0.0044 (13)	0.0170 (14)	0.0012 (13)
C17	0.0366 (17)	0.0481 (19)	0.0443 (16)	0.0017 (13)	0.0074 (13)	−0.0041 (13)
C18	0.0306 (15)	0.0418 (17)	0.0506 (16)	0.0011 (13)	0.0108 (12)	0.0004 (13)
C19	0.056 (2)	0.053 (2)	0.0541 (19)	−0.0043 (16)	0.0184 (15)	0.0009 (15)
C20	0.075 (3)	0.059 (2)	0.078 (2)	−0.008 (2)	0.023 (2)	0.018 (2)
C21	0.073 (3)	0.044 (2)	0.109 (3)	−0.0100 (19)	0.028 (2)	0.010 (2)
C22	0.073 (3)	0.053 (2)	0.083 (3)	−0.0132 (19)	0.003 (2)	−0.012 (2)
C23	0.056 (2)	0.049 (2)	0.0568 (19)	−0.0060 (16)	0.0055 (16)	−0.0004 (15)
C24	0.054 (2)	0.0437 (19)	0.0623 (19)	−0.0003 (16)	0.0217 (16)	0.0017 (15)
O2	0.091 (2)	0.0373 (14)	0.146 (2)	−0.0028 (13)	0.0641 (18)	0.0081 (15)
C25	0.124 (4)	0.053 (3)	0.191 (5)	−0.012 (3)	0.083 (4)	0.011 (3)
C26	0.273 (9)	0.201 (8)	0.157 (7)	−0.137 (7)	0.041 (6)	0.024 (6)
C26'	0.17 (4)	0.070 (19)	0.09 (2)	−0.04 (2)	−0.01 (2)	0.040 (15)
Cl1	0.1111 (10)	0.0425 (6)	0.1552 (11)	−0.0098 (6)	0.0502 (8)	0.0038 (6)
F1	0.136 (2)	0.0720 (15)	0.0778 (14)	0.0048 (14)	0.0615 (15)	−0.0188 (12)
N1	0.100 (3)	0.0414 (18)	0.087 (2)	−0.0051 (17)	0.0263 (19)	−0.0152 (16)
N2	0.0427 (15)	0.0378 (14)	0.0498 (13)	0.0029 (11)	0.0170 (11)	−0.0048 (11)
N3	0.0374 (15)	0.0499 (16)	0.0529 (14)	−0.0005 (12)	0.0179 (12)	−0.0109 (12)
N4	0.0394 (15)	0.0388 (14)	0.0532 (14)	−0.0004 (11)	0.0155 (11)	−0.0074 (11)
N5	0.0359 (13)	0.0388 (14)	0.0428 (12)	0.0002 (10)	0.0086 (10)	−0.0020 (10)
O1	0.0646 (16)	0.0559 (15)	0.0730 (15)	−0.0033 (12)	0.0391 (13)	0.0018 (11)
S1	0.0492 (5)	0.0393 (5)	0.0713 (5)	0.0058 (4)	0.0232 (4)	−0.0017 (4)
S2	0.0431 (5)	0.0595 (6)	0.0667 (5)	−0.0006 (4)	0.0237 (4)	−0.0088 (4)

### Geometric parameters (Å, º)

**Table:**

C1—N1	1.325 (5)	C15—N5	1.401 (3)
C1—C2	1.372 (5)	C16—C24	1.457 (4)
C1—Cl1	1.746 (4)	C16—S1	1.736 (3)
C2—C3	1.379 (5)	C17—N5	1.373 (4)
C2—H2	0.9300	C17—S2	1.664 (3)
C3—C4	1.376 (4)	C17—S1	1.712 (3)
C3—H3	0.9300	C18—C19	1.373 (4)
C4—C5	1.370 (4)	C18—C23	1.381 (4)
C4—C6	1.509 (4)	C18—N5	1.444 (4)
C5—N1	1.334 (4)	C19—C20	1.375 (5)
C5—H5	0.9300	C19—H19	0.9300
C6—N2	1.452 (4)	C20—C21	1.364 (5)
C6—H6A	0.9700	C20—H20	0.9300
C6—H6B	0.9700	C21—C22	1.377 (5)
C7—N2	1.455 (4)	C21—H21	0.9300
C7—H7A	0.9600	C22—C23	1.370 (5)
C7—H7B	0.9600	C22—H22	0.9300
C7—H7C	0.9600	C23—H23	0.9300
C8—N4	1.304 (4)	C24—O1	1.204 (4)
C8—N3	1.354 (4)	C24—O2	1.340 (4)
C8—N2	1.359 (3)	O2—C25	1.464 (4)
C9—C10	1.374 (4)	C25—C26	1.506 (6)
C9—C14	1.383 (4)	C25—C26'	1.529 (9)
C9—N3	1.424 (4)	C25—H25A	0.9700
C10—C11	1.391 (4)	C25—H25B	0.9700
C10—H10	0.9300	C25—H25C	0.9700
C11—C12	1.364 (5)	C25—H25D	0.9700
C11—H11	0.9300	C26—H26A	0.9600
C12—C13	1.361 (5)	C26—H26B	0.9600
C12—F1	1.363 (3)	C26—H26C	0.9600
C13—C14	1.381 (4)	C26'—H26D	0.9600
C13—H13	0.9300	C26'—H26E	0.9600
C14—H14	0.9300	C26'—H26F	0.9600
C15—N4	1.356 (3)	N3—H3A	0.86 (3)
C15—C16	1.371 (4)		
			
N1—C1—C2	124.9 (3)	C19—C20—H20	119.9
N1—C1—Cl1	116.3 (3)	C20—C21—C22	120.0 (3)
C2—C1—Cl1	118.7 (3)	C20—C21—H21	120.0
C1—C2—C3	117.0 (3)	C22—C21—H21	120.0
C1—C2—H2	121.5	C23—C22—C21	120.6 (3)
C3—C2—H2	121.5	C23—C22—H22	119.7
C4—C3—C2	120.5 (3)	C21—C22—H22	119.7
C4—C3—H3	119.8	C22—C23—C18	118.8 (3)
C2—C3—H3	119.8	C22—C23—H23	120.6
C5—C4—C3	116.6 (3)	C18—C23—H23	120.6
C5—C4—C6	120.7 (3)	O1—C24—O2	123.9 (3)
C3—C4—C6	122.7 (3)	O1—C24—C16	126.4 (3)
N1—C5—C4	125.3 (3)	O2—C24—C16	109.7 (3)
N1—C5—H5	117.3	C24—O2—C25	117.5 (3)
C4—C5—H5	117.3	O2—C25—C26	110.1 (4)
N2—C6—C4	113.7 (2)	O2—C25—C26'	109.7 (6)
N2—C6—H6A	108.8	C26—C25—C26'	139.1 (7)
C4—C6—H6A	108.8	O2—C25—H25A	109.6
N2—C6—H6B	108.8	C26—C25—H25A	109.6
C4—C6—H6B	108.8	C26'—C25—H25A	46.7
H6A—C6—H6B	107.7	O2—C25—H25B	109.6
N2—C7—H7A	109.5	C26—C25—H25B	109.6
N2—C7—H7B	109.5	C26'—C25—H25B	64.3
H7A—C7—H7B	109.5	H25A—C25—H25B	108.2
N2—C7—H7C	109.5	O2—C25—H25C	110.2
H7A—C7—H7C	109.5	C26—C25—H25C	69.1
H7B—C7—H7C	109.5	C26'—C25—H25C	105.1
N4—C8—N3	126.7 (3)	H25A—C25—H25C	137.6
N4—C8—N2	117.6 (3)	H25B—C25—H25C	43.4
N3—C8—N2	115.5 (3)	O2—C25—H25D	110.2
C10—C9—C14	120.3 (3)	C26—C25—H25D	42.0
C10—C9—N3	118.7 (3)	C26'—C25—H25D	113.4
C14—C9—N3	121.0 (3)	H25A—C25—H25D	70.5
C9—C10—C11	119.8 (3)	H25B—C25—H25D	137.7
C9—C10—H10	120.1	H25C—C25—H25D	108.1
C11—C10—H10	120.1	C25—C26—H25C	38.0
C12—C11—C10	118.4 (3)	C25—C26—H25D	39.6
C12—C11—H11	120.8	H25C—C26—H25D	75.8
C10—C11—H11	120.8	C25—C26—H26A	109.5
C13—C12—F1	118.9 (3)	H25C—C26—H26A	139.1
C13—C12—C11	122.9 (3)	H25D—C26—H26A	84.9
F1—C12—C11	118.2 (3)	C25—C26—H26B	109.5
C12—C13—C14	118.5 (3)	H25C—C26—H26B	106.4
C12—C13—H13	120.7	H25D—C26—H26B	90.2
C14—C13—H13	120.7	C25—C26—H26C	109.5
C13—C14—C9	120.0 (3)	H25C—C26—H26C	75.5
C13—C14—H14	120.0	H25D—C26—H26C	148.8
C9—C14—H14	120.0	C25—C26'—H26D	109.5
N4—C15—C16	133.7 (3)	C25—C26'—H26E	109.5
N4—C15—N5	115.2 (3)	H26D—C26'—H26E	109.5
C16—C15—N5	111.0 (2)	C25—C26'—H26F	109.5
C15—C16—C24	127.9 (3)	H26D—C26'—H26F	109.5
C15—C16—S1	111.4 (2)	H26E—C26'—H26F	109.5
C24—C16—S1	120.7 (2)	C1—N1—C5	115.6 (3)
N5—C17—S2	127.1 (2)	C8—N2—C6	120.3 (2)
N5—C17—S1	109.4 (2)	C8—N2—C7	123.6 (2)
S2—C17—S1	123.53 (18)	C6—N2—C7	115.4 (2)
C19—C18—C23	120.9 (3)	C8—N3—C9	126.4 (3)
C19—C18—N5	120.1 (3)	C8—N3—H3A	116 (2)
C23—C18—N5	119.0 (3)	C9—N3—H3A	116 (2)
C18—C19—C20	119.4 (3)	C8—N4—C15	125.9 (2)
C18—C19—H19	120.3	C17—N5—C15	115.7 (2)
C20—C19—H19	120.3	C17—N5—C18	122.0 (2)
C21—C20—C19	120.3 (3)	C15—N5—C18	122.2 (2)
C21—C20—H20	119.9	C17—S1—C16	92.42 (14)
			
N1—C1—C2—C3	−1.2 (7)	C16—C24—O2—C25	−179.9 (3)
Cl1—C1—C2—C3	179.3 (3)	C24—O2—C25—C26	−99.3 (6)
C1—C2—C3—C4	0.3 (6)	C24—O2—C25—C26'	90.2 (15)
C2—C3—C4—C5	0.7 (6)	C2—C1—N1—C5	0.9 (6)
C2—C3—C4—C6	−178.9 (4)	Cl1—C1—N1—C5	−179.5 (3)
C3—C4—C5—N1	−1.0 (6)	C4—C5—N1—C1	0.2 (6)
C6—C4—C5—N1	178.7 (4)	N4—C8—N2—C6	−4.5 (4)
C5—C4—C6—N2	140.5 (3)	N3—C8—N2—C6	−180.0 (2)
C3—C4—C6—N2	−39.9 (4)	N4—C8—N2—C7	165.5 (3)
C14—C9—C10—C11	0.8 (5)	N3—C8—N2—C7	−9.9 (4)
N3—C9—C10—C11	178.3 (3)	C4—C6—N2—C8	98.3 (3)
C9—C10—C11—C12	2.1 (5)	C4—C6—N2—C7	−72.6 (3)
C10—C11—C12—C13	−2.4 (6)	N4—C8—N3—C9	−7.1 (5)
C10—C11—C12—F1	177.2 (3)	N2—C8—N3—C9	167.9 (3)
F1—C12—C13—C14	−179.8 (3)	C10—C9—N3—C8	135.3 (3)
C11—C12—C13—C14	−0.2 (5)	C14—C9—N3—C8	−47.2 (4)
C12—C13—C14—C9	3.1 (5)	N3—C8—N4—C15	−26.7 (4)
C10—C9—C14—C13	−3.4 (5)	N2—C8—N4—C15	158.4 (3)
N3—C9—C14—C13	179.1 (3)	C16—C15—N4—C8	−43.7 (5)
N4—C15—C16—C24	1.6 (5)	N5—C15—N4—C8	142.1 (3)
N5—C15—C16—C24	176.0 (3)	S2—C17—N5—C15	176.7 (2)
N4—C15—C16—S1	−177.2 (3)	S1—C17—N5—C15	−2.4 (3)
N5—C15—C16—S1	−2.8 (3)	S2—C17—N5—C18	−0.8 (4)
C23—C18—C19—C20	−0.3 (5)	S1—C17—N5—C18	−179.93 (19)
N5—C18—C19—C20	178.2 (3)	N4—C15—N5—C17	178.9 (2)
C18—C19—C20—C21	0.5 (6)	C16—C15—N5—C17	3.4 (3)
C19—C20—C21—C22	−0.5 (6)	N4—C15—N5—C18	−3.5 (4)
C20—C21—C22—C23	0.4 (6)	C16—C15—N5—C18	−179.0 (2)
C21—C22—C23—C18	−0.3 (6)	C19—C18—N5—C17	72.3 (4)
C19—C18—C23—C22	0.3 (5)	C23—C18—N5—C17	−109.1 (3)
N5—C18—C23—C22	−178.3 (3)	C19—C18—N5—C15	−105.2 (3)
C15—C16—C24—O1	−0.9 (6)	C23—C18—N5—C15	73.5 (4)
S1—C16—C24—O1	177.8 (3)	N5—C17—S1—C16	0.6 (2)
C15—C16—C24—O2	−179.7 (3)	S2—C17—S1—C16	−178.58 (19)
S1—C16—C24—O2	−1.0 (4)	C15—C16—S1—C17	1.3 (2)
O1—C24—O2—C25	1.3 (5)	C24—C16—S1—C17	−177.5 (3)

### Hydrogen-bond geometry (Å, º)

**Table:**

*D*—H···*A*	*D*—H	H···*A*	*D*···*A*	*D*—H···*A*
N3—H3*A*···S2^i^	0.86 (3)	2.65 (3)	3.443 (3)	153 (3)
C7—H7*B*···O1^ii^	0.96	2.51	3.282 (4)	138

Symmetry codes: (i) *x*−1, *y*, *z*; (ii) −*x*, −*y*+1, −*z*+2.
